# Fatigue fracture of a DBS extension cable: a pictorial review

**DOI:** 10.1308/rcsann.2025.0012

**Published:** 2025-04-08

**Authors:** Q Al Banna, H Lalgudi Srinivasan, JC Knight, M Samuel, I Bodi, K Ashkan

**Affiliations:** King’s College Hospital NHS Foundation Trust, UK

**Keywords:** Deep brain stimulation, Hardware complication in DBS, Long-term DBS complication, Extension cable fracture, Essential tremor

## Abstract

Non-deep brain stimulation (DBS) lead hardware complications are quite uncommon. They are observed more with tremor and dystonia patients due to constant strain on the neck region. However, occurrence of such complication over a two-decade period has not been reported. Twenty years after DBS implantation, a patient presented with a wear and tear fracture in the extension cable, which we describe as a fatigue fracture of the extension cable. Delayed hardware complications following DBS implantation is an under-reported entity due to follow-up compliance over the long term. Reporting such complications is essential to understand the durability of the hardware, and to anticipate and manage implant failure.

## Background

For medically refractory tremors, VIM (ventralis intermedius nucleus)-targeted DBS (deep brain stimulation) is the gold standard surgical intervention, with excellent results over the long term. Apart from lead dysfunction, other hardware complications are less commonly recognised and reported. Over the long term, the non-lead component (extension cable) of a DBS implant is more prone to wear and tear like an intracranially placed lead. Here, we describe one such hardware failure, the mechanism and the management strategy.

## Case history

**Figure 1:**
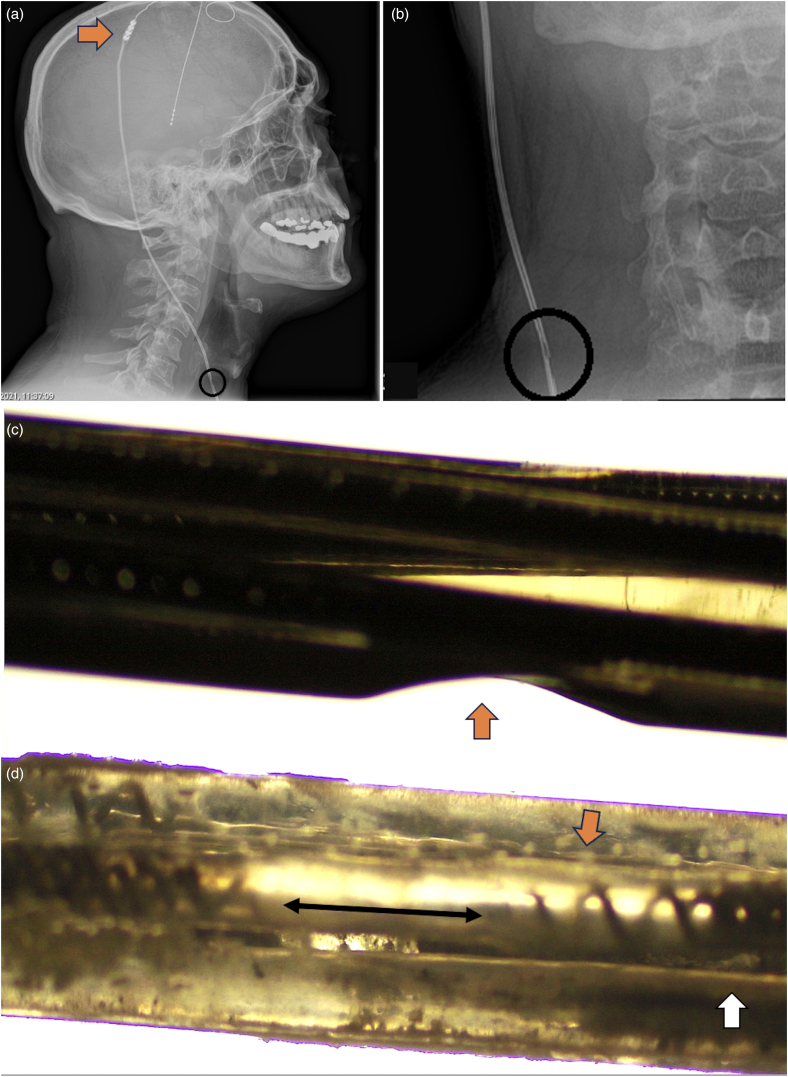
(a) Lateral view x-ray depicting the breach in extension cable (black circle) and parietal placement of lead-extension connector (orange arrow). (b) Magnified antero-posterior view of x-ray depicting breach in extension cable (black circle). (c) Optical microscope view (×40 magnification with ×4 objective) showing breach in insulation coating of extension cable (orange arrow) from the surgical specimen. (d) Optical microscope view (×40 magnification with ×4 objective) showing break in the helical wires (double-headed arrow), deformation of helical wires (orange arrow) and normal configuration of helical wires (white arrow).

A 37-year-old man with unilateral (left side) action and postural tremor underwent VIM nucleus DBS on his right side in 2001 (Medtronic, Minneapolis, MN, USA). He had good clinical improvement and had been on regular clinical follow-up. He had undergone three battery revisions over two decades with last revision in 2020 (the first two revisions were Activa PC, Medtronic, with the last revision being Activa RC, Medtronic, based on patient preference). Six months after the last battery change, the patient noticed a gradual worsening in his tremors. On unipolar interrogation, high impedance (>2,000Ω) was observed in contact 1 and 2, and normal impedance in other contacts (Contact 0, 925Ω; Contact 3, 662Ω). The patient then complained of a shock-like sensation in his neck and a need for frequent battery recharging. An x-ray examination revealed an unusual breach in the extension cable (Figure 1a and b). He underwent extension cable replacement, after which he noticed improvement in his symptoms (now at 24 months follow-up). A high-definition microscopic examination showed a breach in the insulation sheath (Figure 1c) and in the helical loop (Figure 1d).

## Discussion

Our patient had excellent long-term clinical control of his tremor until the event described above. Fracture in a DBS system results in high impedance as noted in our patient.^1^ However, a shock-like sensation with frequent drainage of battery (as seen in our patient) usually indicates a short-circuit.^1^ On a plain x-ray image, a breach in the extension cable was observed (Figure 1a and b). Microscope views showed a break in the helical wire and insulation breach in the extension cable (Figure 1c and d), which have not been documented in literature. This unique pictorial representation describes a fatigue fracture in a DBS extension cable, which has not been reported previously.

In a systematic review, long-term hardware complications were observed in 11.75% patients.^2^ In this latter review, among 9,000 patients, lead fracture was the third most common complication (131 patients, 1.46%). However, only 66 patients (0.73%) had a fracture in the nonlead component.^2^ Due to repetitive cervical movements, DBS hardware fracture is observed more often in patients with dystonia and tremor.^2,3^ Sudden loss in clinical effect with high impedance indicates a breach in the DBS system.^2^ Length of extension cable and lead–extension connector placement may contribute to hardware fracture.^3,4^ A short extension cable (40cm) with postauricular or cervical connector placement places strain on the lead during each neck movement and may cause lead fracture.^4,5^ However, a short extension cable (40cm) with parietal connector placement causes strain on the extension cable.^2,4^ Our patient had a 40cm extension cable with connector placed at the parietal level (Figure 1a). Hence, during each neck movement there was additional strain on the extension cable. In general extension cables have greater resistance to strain than the lead.^5^ Like a lead, an extension cable adopts a helical structure with high tolerance to stress (fatigue life of >400,000 cycles).^3^ In an experimental study, deformation of the helical structure in a lead reduced the fatigue life exponentially (20% deformation equals 20-fold reduction).^3^ However, normal material fatigue over the past 20 years along with parietal placement of connector, repetitive neck movements and possible growth (in a child) could all contribute to the deformation and weakening of the extension cable. This could explain the unusual fracture in our patient’s extension cable, representing an under-recognised very late complication of DBS.^5^

## Conclusion

This unique pictorial representation describes a fatigue fracture in a DBS extension cable – an important late complication of DBS that will need to be recognised as more and more patients with DBS enter their later decades postimplantation.
